# Efficacy and safety of double-dose statin monotherapy versus moderate-intensity statin combined with ezetimibe dual therapy in diabetic patients: a systematic review and meta-analysis of randomized controlled trials

**DOI:** 10.1097/XCE.0000000000000315

**Published:** 2024-10-25

**Authors:** Aman Goyal, Muhammad Daoud Tariq, Hritvik Jain, Abhigan Babu Shrestha, Laveeza Fatima, Romana Riyaz, Hritik Raj Yadav, Darsh Safi, Abdul Qahar K. Yasinzai, Rozi Khan, Amir Humza Sohail, Mohamed Daoud, Abu Baker Sheikh

**Affiliations:** Department of Internal Medicine, a Seth GS Medical College and KEM Hospital, Mumbai, India; b Foundation University Medical College, Islamabad, Pakistan; c All India Institute of Medical Sciences-Jodhpur, Jodhpur, Rajasthan, India; d M Abdur Rahim Medical College, Dinajpur, Bangladesh; e Allama Iqbal Medical College, Lahore, Pakistan; f Shadan Institute of Medical Sciences and Research, Hyderabad, India; g M Abdur Rahim Medical College, Dinajpur, Bangladesh; h Bolan Medical College, Quetta, Pakistan; i Medical University of South Carolina, Florence, South Carolina, USA; j Department of Surgery, University of New Mexico Health Sciences, Albuquerque, New Mexico, USA; k Department of Internal Medicine, Bogomolets National Medical University, Kyiv, Ukraine; l Department of Internal Medicine, University of New Mexico Health Sciences, Albuquerque, New Mexico, USA

**Keywords:** cardiovascular disease, double-dose statin therapy, dyslipidemia, high-intensity statin therapy, ezetimibe, moderate-intensity statin, type 2 diabetes mellitus

## Abstract

Cardiovascular disease is a leading cause of mortality, especially in individuals with type 2 diabetes mellitus and dyslipidemia. Despite adequate statin therapy, some patients fail to achieve the target low-density lipoprotein-cholesterol levels. Trials have compared doubling the statin dose with the addition of ezetimibe. A systematic literature search was performed using various databases. Forest plots were constructed for pooled analysis with statistical significance set at *P* < 0.05. Seven trials were included. Monotherapy showed no significant difference compared with dual therapy for low-density lipoprotein-cholesterol levels [mean difference (MD): −5.03; *P* = 0.37], high-density lipoprotein-cholesterol levels (MD: 0.01; *P* = 0.95), total cholesterol (MD: −2.38; *P* = 0.66), and triglycerides (MD: 5.37; *P* = 0.67) at follow-up compared to baseline. Monotherapy significantly reduced serious clinical adverse events (risk ratio: 0.21; *P* = 0.04), with no difference in treatment-related adverse effects, discontinuation due to treatment-related or overall adverse events.

## Graphical abstract

**Figure:**
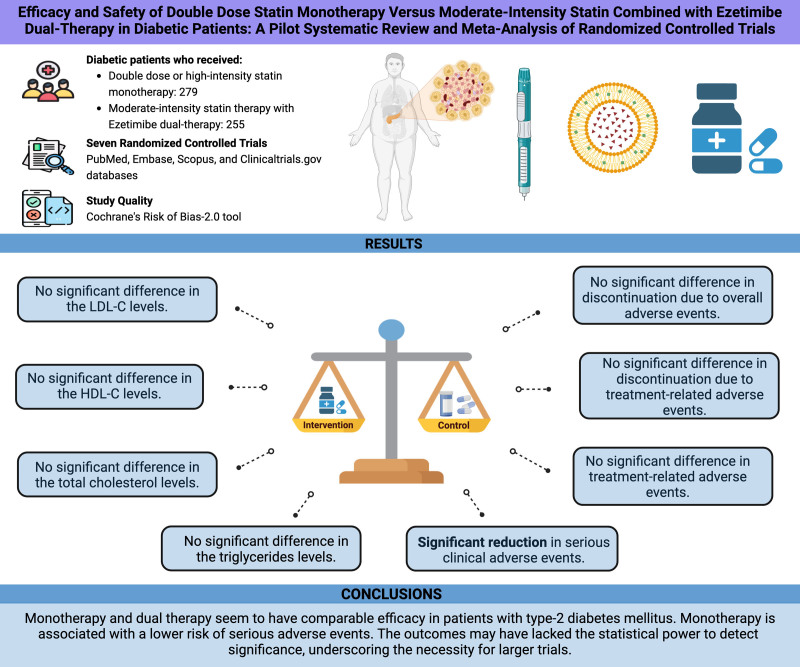

## Introduction

Cardiovascular disease (CVD) is a prominent global cause of mortality. Type 2 diabetes mellitus (T2DM) and dyslipidemia present significant risk for cardiovascular outcomes, including coronary heart disease, stroke, and peripheral artery disease [1,2]. These illnesses contribute to higher mortality rates and reduced quality of life [1,2]. For the management of CVDs, several interventions, including dietary, lifestyle, and pharmaceutical interventions, have been suggested [3–5]. Statins are considered the initial therapeutic intervention for CVD in patients with elevated cholesterol levels or an elevated risk of coronary heart disease. Their efficacy in reducing cholesterol levels and providing protection against coronary heart disease has been well-established in previous studies [6]. However, certain individuals may fail to achieve their target low-density lipoprotein-cholesterol (LDL-C) levels despite receiving high-intensity statin therapy, leading to an increased risk of liver and muscle-related side effects owing to drug metabolism and clearance [7].

The increased potential for adverse effects with dose escalation is a significant concern, prompting the consideration of lower-dose statin therapy or alternative treatment regimens for patients with susceptibility to muscle or liver-related complications. Given the increased risk associated with double-dose or high-intensity statin regimens, the inclusion of ezetimibe as adjunct therapy alongside statins is strongly recommended [8]. Ezetimibe, a nonstatin drug, inhibits cholesterol absorption in the small intestine through its interaction with Niemann-Pick C1-like protein 1. This mechanism results in a significant reduction in LDL-C levels when administered alongside statin [9]. In a comprehensive long-term investigation, it was discovered that the combined use of moderate-intensity statins and ezetimibe demonstrated comparable efficacy to high-dose statin therapy in terms of major adverse cardiovascular event outcomes and reduction of LDL-C levels among patients diagnosed with atherosclerotic CVD [10]. Multiple clinical trials have compared double-dose statin therapy with a combination of ezetimibe and statins at lower or moderate intensities. These findings indicate that combining ezetimibe with statins leads to significantly lower LDL-C levels than double-dose statin therapy alone [11–13]. However, this comparison has not been well defined in the diabetic population, with conflicting results.

Our pilot systemic review and meta-analysis aimed to compare the safety and efficacy of double-dose statin monotherapy versus combination therapy with moderate-dose statin and ezetimibe in participants with T2DM.

## Materials and methodology

The meta-analysis was performed in accordance with the procedures suggested by the Cochrane Collaboration [14] and reported following the Preferred Reporting Items for Systematic Review and Meta-Analysis Statement (2020) [15]. The study protocol was registered with the PROSPERO International Prospective Register of Systematic Reviews with the identification number (CRD42024499922).

### Data sources and search strategy

An electronic search was conducted across various databases, including *MEDLINE* via *PubMed, EMBASE*, *clinicaltrial.gov*, and *SCOPUS*, from inception to February 2024. This comprehensive search encompassed all randomized controlled trials (RCTs) to date. We also searched the conference proceedings of the Journal of American College of Cardiology, Circulation Research, and the European Heart Journal. The aim of our study was to compare the effectiveness of monotherapy with double-dose or high-intensity statin therapy versus dual therapy with moderate-intensity statin therapy and ezetimibe in hypercholesterolemic patients with T2DM. No restrictions were placed on language or timeframe during the search process. To ensure thoroughness, manual searches of the reference lists were conducted. The search strategy involved the utilization of predefined Medical Subject Headings terms in conjunction with Boolean operators ‘AND’ and’ OR’.

The detailed search strategy is available in the Online Supplementary File, Supplemental digital content 2, http://links.lww.com/CAEN/A63.

### Study assessment

Two investigators (M.D.T. and A.G.) independently assessed the titles and abstracts of potential studies to determine eligibility, excluding duplicates or those that did not meet the predefined inclusion criteria. Subsequently, the full texts of the selected articles were scrutinized to exclude studies that did not meet the predetermined eligibility criteria. Detailed examination of the included articles was performed to extract baseline characteristics, such as authorship, publication year, study design, follow-up duration, population size, comorbidities, and other pertinent factors. Any discrepancies were resolved through a discussion initiated by a third reviewer to achieve a consensus (A.B.S.). No restrictions were placed on the sample size or follow-up duration.

### Eligibility criteria

#### Inclusion criteria

The inclusion criteria were based on the PICOS format for systematic reviews and meta-analyses, with population (P) being patients with T2DM, intervention (I) being monotherapy with double-dose or high-intensity statin therapy, control (C) being dual therapy with moderate-intensity statins and ezetimibe, and outcomes (O) with various parameters discussed in the subsequent paragraphs. Only RCTs were included for statistical rigor.

#### Exclusion criteria

The exclusion criteria were: (1) studies that fit into any of the following categories: case reports and series, literature reviews, systematic reviews, meta-analyses, letters to editors, or observational studies were disqualified; further exclusion criteria included: (2) patients with a history of recent major cardiovascular events or uncontrolled hypertension, (3) patients with active inflammatory or infectious disease, malignancy, a history of hypersensitivity to any of the drugs involved, severe liver or renal dysfunction, and insulin-dependent diabetes; (4) pregnant women, women who might be pregnant, and breastfeeding women; (5) studies using animal models; and (6) studies lacking adequate clinical data pertinent to the outcomes being studied.

### Data extraction and quality assessment

Two researchers (M.D.T. and A.G.) extracted the data from the included studies into a prepiloted Microsoft Excel spreadsheet. In case of disagreement, an independent researcher reviewed the data (A.B.S.). The extracted information included the first author’s name, publication year, country of origin, study design, sample size (intervention or control groups), and reported outcomes. Quality evaluation was conducted using the modified Cochrane Collaboration risk-of-bias 2.0 tool specifically designed for RCTs [16].

### Endpoint definition

The efficacy endpoints were changes in the LDL-C, high-density lipoprotein-cholesterol (HDL-C), total cholesterol, triglyceride levels, and changes in hemoglobin A1c (HBA1c), homeostatic model assessment (HOMA)-IR, and HOMA-B levels. The safety endpoints included treatment-related adverse effects, discontinuation of therapy secondary to treatment-related adverse effects, discontinuation due to adverse events, and serious adverse clinical events. Prespecified reasons for discontinuation were used, which included the following: myopathy, two consecutive increases in levels of creatine kinase >10 times the upper limit of normal with or without symptoms, or greater than five times the upper limit of normal with symptoms; persistent increases greater than three times the upper limit of normal in alanine aminotransferase; or two consecutive triglyceride values of >11.3 mmol/l (1000 mg/dl).

### Data synthesis

The extracted data were pooled and analyzed using Review Manager Version 5.4 (Cochrane Collaboration) by two authors (A.G. and A.B.S.). For continuous outcomes, the mean difference (MD) between follow-up and baseline values was used to pool data. For dichotomous outcomes, the risk ratio (RR) was used as an effect measure. The Mantel-Haenszel random-effects meta-analyses were used for all dichotomous outcomes, while Generic Invariance random-effects models were used for continuous outcomes. Higgins *I*^2^ statistics were used to evaluate statistical heterogeneity; a value of <25% was considered as low heterogeneity, 25–75% as moderate heterogeneity, and >75% as high heterogeneity. Sensitivity analysis was used to explore the influential studies. Visual inspection of the funnel plots was done to assess for publication bias.

## Results

### Study selection and characteristics

**Fig. 1:**
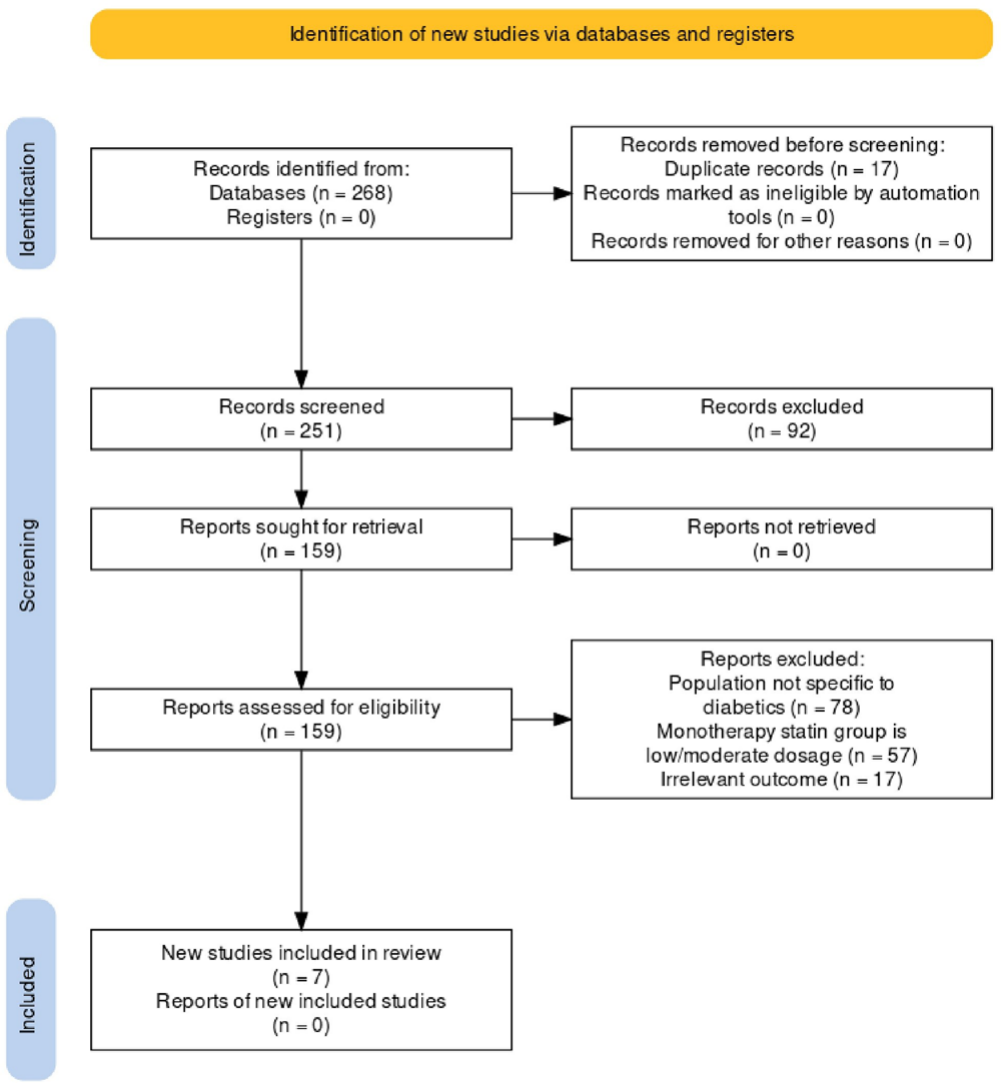
The 2020 Preferred Reporting Items for Systematic Reviews and Meta-Analyses Flowchart.

A total of 268 records were identified from various databases. After removing 17 duplicates, 251 studies were further screened. A total of 159 studies were assessed using their full texts after removing studies that did not meet our inclusion criteria. Further text screening revealed 78 studies that did not report outcomes specific to the diabetic population (n = 78), 57 studies utilized low or moderate doses of statin therapy in the intervention/monotherapy group, and 17 studies reported irrelevant outcomes. Seven RCTs [17–23] were included in the final analysis, comprising 279 patients in the monotherapy group (receiving double-dose or high-intensity statin therapy) and 255 patients in the dual therapy group (receiving moderate-intensity statin with ezetimibe therapy). This selection process is depicted in the Preferred Reporting Items for Systematic Review and Meta-Analysis flowchart, Fig. 1. The specific drugs used for each group at their respective doses are listed in Table 1. In the monotherapy and dual therapy groups, the average age and BMI were 64.4 and 64.56 years, and 29 and 28.72 kg/m², respectively. The mean follow-up period was 15.7 weeks. The baseline characteristics of the patients are summarized in Table 2.

**Table 1:** Drug name, dose, and sample size of included studies

Study ID	Study Design	Total sample size	Monotherapy drug, dose, sample size	Dual therapy drug, dose, sample size
Bardini *et al* [21].	Randomized controlled trial	93	Simvastatin, 40 mg, *N* = 51	Ezetimibe 10 mg plus simvastatin 20 mg, *N* = 42
Gaudiani *et al* [22].	Randomized controlled trial	214	Simvastatin, 40 mg, *N* = 104	Ezetimibe 10 mg plus simvastatin 20 mg, *N* = 110
Kawagoe *et al* [17].	Randomized controlled trial	24	Fluvastatin, 60 mg, *N* = 12	Ezetimibe 10 mg and Fluvastatin 20 mg, *N* = 12
Moon *et al* [23].	Randomized controlled trial	99	Rosuvastatin, 20 mg, *N* = 51	Ezetimibe 10 mg plus Rosuvastatin 10 mg, *N* = 48
Sakamoto *et al* [24].	Randomized controlled trial	109	Atorvastatin (20 mg) or pitavastatin (2 mg) of, *N* = 56	Ezetimibe 10 mg plus Atorvastatin (10 mg) or Pitavastatin (1 mg), *N* = 53
Settergren *et al* [18].	Randomized controlled trial	43	Simvastatin, 80 mg, *N* = 22	Ezetimibe 10 mg plus simvastatin 10 mg, *N* = 21
Uemura *et al* [20].	Randomized controlled trial	39	Atorvastatin, 20 mg, *N* = 20	Ezetimibe 10 mg plus Atorvastatin 10 mg, *N* = 19

**Table 2:** Baseline characteristics of patients

Study	Age (in years)		Sample size		Hypertension		LDL-C		HDL-C		HBA1c		Non-HDL		TG	
	Mono	Dual	Mono	Dual	Mono	Dual	Mono	Dual	Mono	Dual	Mono	Dual	Mono	Dual	Mono	Dual
Bardini *et al* [21].	64	65	50	37	32	30	3.2 (0.5)	3.3 (0.5)	1.1 (0.3)	1.2 (0.3)	7.4 (0.8)	7.5 (0.7)	NR	NR	1.6 (0.7)	1.6 (0.7)
Gaudiani *et al* [22].	58.3	57.8	107	103	NR	NR	2.37 (0.63)	2.43 (0.74)	1.27 (0.28)	1.23 (0.28)	7.3 (1.1)	7.3 (1.3)	3.08 (0.80)	3.23 (1.02)	1.71 (1.25)	1.69 (1.30)
Kawagoe *et al* [17].	65.1 (7.2)	64.2 (7.2)	12	12	NR	NR	154 (26)	164 (33)	55.4 (16)	57.2 (18)	6.17 (0.86)	6.50 (0.62)	NR	NR	115 (28.9)	128 (57.9)
Moon SJ *et al* [23].	61.16 (7.0)	61.88 (6.4)	51	48	29	28	131.36 (29.49)	140.54 (31.63)	46.57 (10.59)	50.21 (11.0)	7.39 (1.02)	7.38 (0.88)	152.61 (32.40)	158.77 (34.52)	202.51 (134.19)	189.04 (113.51)
Sakamoto *et al*.	62.6 (9.5)	61.7 (11.1)	56	53	NR	NR	132 (24)	126 (21)	54.7 (9.6)	56.7 (15.2)	7.26 (0.97)	7.24 (0.65)	164 (25)	155 (27)	162 (88)	147 (95)
Settergren *et al* [18].	70	74	20	19	150	60	3.0 (IQR: 2.5–3.7)	3.1 (IQR: 2.8–3.4)	0.9 (IQR 0.8–1.2)	1.0 (IQR 0.9–1.2)	5.5 (IQR 5.0–7.2)	6.2 (IQR 4.3–7.1)	NR	NR	1.8 (IQR 1.2–2.6)	1.4 (IQR 1.0–2.2)
Uemura *et al* [20].	67.8 (9.7)	NR	19	20	36	NR	111.6 (IQR: 16.6)	NR	46.7 (10.2)	NR	5.70 (0.61)	NR	NR	NR	124.3 (55.9)	NR

All values in parenthesis are standard deviations unless.

Dual, dual therapy group; HBA1c, hemoglobin A1c; HDL-C, high-density lipoprotein-cholesterol; IQR, interquartile range for median values; LDL-C, low-density lipoprotein-cholesterol; Mono, monotherapy group; NR, not reported, RCT, randomized controlled trials.

### Endpoints

Studies were allocated for statistical analysis based on the data availability from each study. For the efficacy analysis, Kawagoe *et al*. [17], Settergren *et al*. [18], Hwang *et al*. [19] and Uemura *et al*. [20] were eligible, whereas outcomes for the safety profile were reported by Bardini *et al*. [21], Gaudiani *et al*. [22], and Moon *et al*. [23].

### Efficacy analysis

**Fig. 2:**
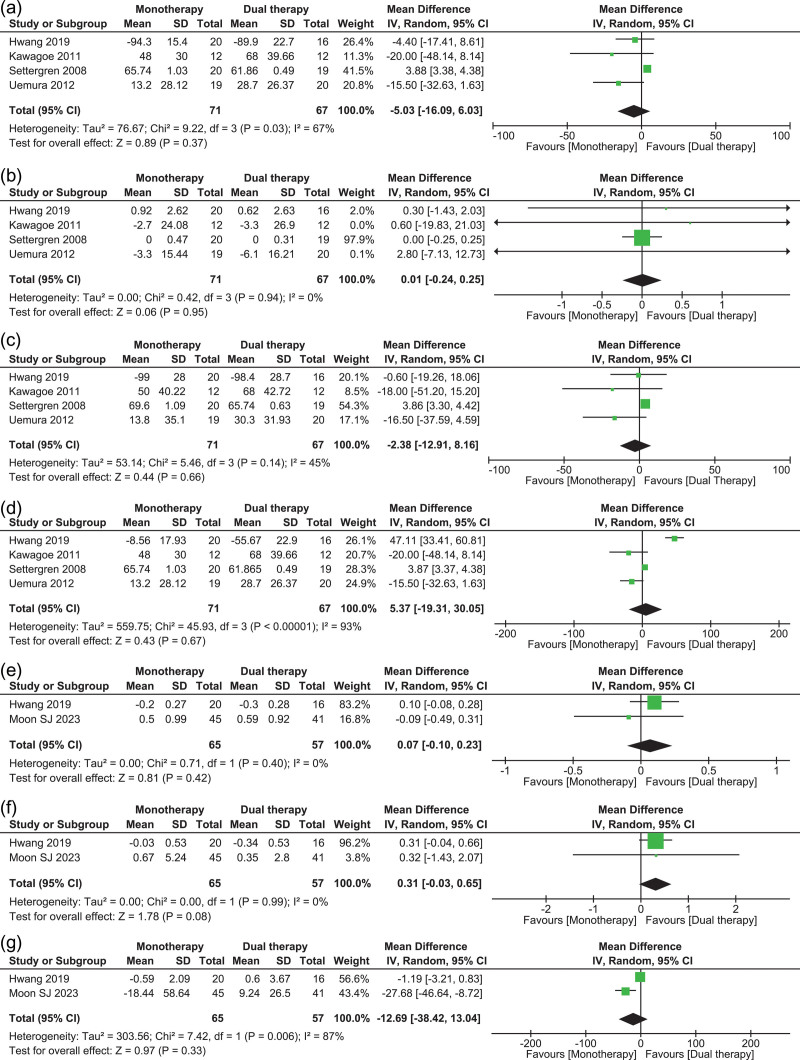
Pooled analysis illustrating the efficacy outcomes of double-dose statin compared to moderate-intensity statin therapy combined with ezetimibe therapy. The mean difference (MD) and risk ratio (RR) with their 95% confidence interval (CI) are displayed using a logarithmic scale, with the box size scaling in accordance with the sample size. The diamond symbolizes the combined or overall effect.

Four studies [17–20] reported the outcomes of all lipid level-related efficacy analyses. No statistically significant difference was found between the monotherapy and dual therapy groups in the change in LDL-C levels at follow-up versus baseline [MD, −5.03; 95% confidence interval (CI), −16.09 to 6.03; *I*^2^ = 67%; *P* = 0.37] (Fig. 2a). Moderate heterogeneity was reported among the outcomes. Leave-one-out sensitivity analysis identified Settergren *et al*. [18] as the influential study. Upon its removal, the heterogeneity decreased to 0%.

Analysis of changes in HDL-C levels showed no statistical difference between the two groups (MD, 0.01; 95% CI, −0.24 to 0.25; *I*^2^ = 0%; *P* = 0.95) (Fig. 2b). No heterogeneity was reported among the outcomes. No statistical difference was found between the monotherapy and dual therapy groups in the reduction of total cholesterol (MD, −2.38; 95% CI, −12.91 to 8.16; *I*^2^ = 45%; *P* = 0.66) (Fig. 2c), with moderate heterogeneity noted between the studies. Upon performing leave-one-out sensitivity analysis, the removal of Settergren *et al*. [18] resulted in no heterogeneity among the outcomes (*I*^2^ = 0%). Finally, no significant difference was noted between the monotherapy and dual therapy groups for changes in triglyceride levels (MD, 5.37; 95% CI, −19.31 to 30.05; *I*^2^ = 93%; *P* = 0.67) (Fig. 2d). High heterogeneity was reported among the studies, which could not be resolved with sensitivity analysis. Sensitivity analysis revealed that the study by Hwang *et al*. [19] was influential, and its removal reduced the heterogeneity to a ‘moderate’ level, with an *I*^2^ value of 74%.

A pooled analysis of two studies [19,23] for change in HBA1c levels reported no statistical difference between the monotherapy and dual therapy groups (MD, 0.07; 95% CI, −0.10 to 0.23; *I*^2^ = 0%; *P* = 0.42) (Fig. 2e). No heterogeneity was reported among the outcomes. The pooled analysis of the same two studies [19,23] showed no statistically significant difference between the two groups in terms of changes in HOMA-IR (MD, 0.31; 95% CI, −0.03 to 0.65; *I*^2^ = 0%; *P* = 0.08) (Fig. 2f) and HOMA-B (MD, −12.69; 95% CI, −38.42 to 13.04; *I*^2^ = 87%; *P* = 0.33) (Fig. 2-G) levels. High heterogeneity was observed among the studies regarding the outcome of HOMA-B levels.

### Safety analysis

**Fig. 3:**
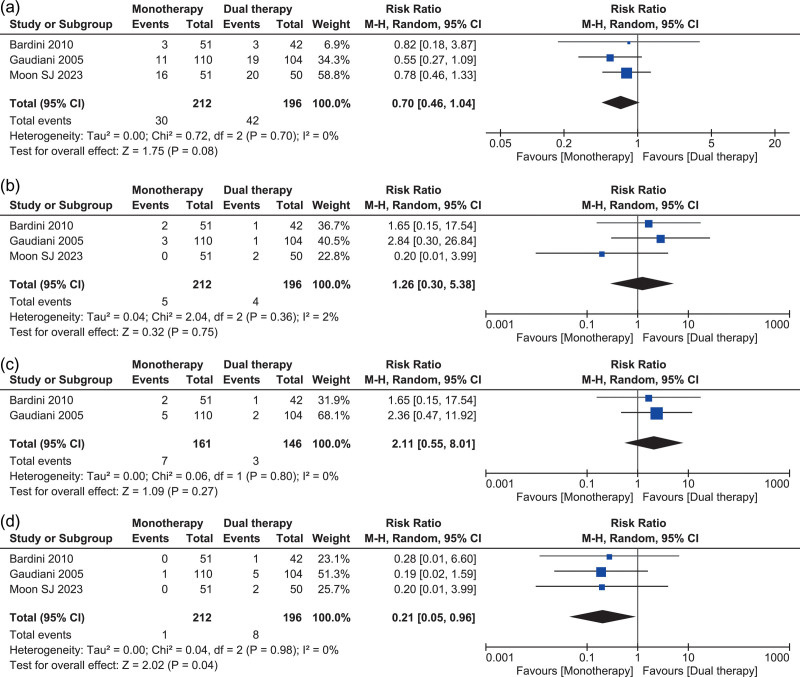
Individual pooled analysis illustrating the safety profile of double-dose statin compared to moderate-intensity statin therapy combined with ezetimibe therapy. The mean difference and risk ratio (RR) with their 95% confidence interval (CI) are displayed using a logarithmic scale, with the box size scaling in accordance with the sample size. The diamond symbolizes the combined or overall effect.

Three studies [21–23] reported all safety outcomes. Monotherapy had a lower risk of treatment-related adverse effects than dual therapy, although this difference was not statistically significant (RR, 0.70; 95% CI, 0.46–1.04; *I*^2^ = 0%; *P* = 0.08) (Fig. 3a). No heterogeneity was observed among the studies. Similarly, when evaluating discontinuation due to treatment-related adverse events, there was no statistical difference between the two groups (RR, 1.26; 95% CI, 0.30–5.38; *I*^2^ = 2%; *P* = 0.75) (Fig. 3b). Low heterogeneity was observed among the studies. For the outcome of discontinuation due to overall adverse events, monotherapy trended towards a higher risk without reaching statistical significance (RR, 2.11; 95% CI, 0.55–8.01; *I*^2^ = 0%; *P* = 0.27) (Fig. 3c). No heterogeneity was observed among the studies. Conversely, concerning serious clinical adverse events, monotherapy demonstrated a statistically significant lower risk than dual therapy (RR, 0.21; 95% CI, 0.05–0.96; *I*^2^ = 0%; *P* = 0.04) (Fig. 3d). No heterogeneity was observed among the studies for this outcome.

### Risk of bias and publication bias assessment

The risk of bias was ‘low’ for most of the included studies. There were ‘some concerns’ for Moon *et al*. [23], Hwang *et al*. [19], and Uemura *et al*. [20] (online Supplementary Figure 4, Supplemental digital content 2, http://links.lww.com/CAEN/A63).

On visual inspection of funnel plots (online Supplementary Figure 5, Supplemental digital content 2, http://links.lww.com/CAEN/A63), symmetrical appearance demonstrated no to low risk of publication bias.

## Discussion

To the best of our knowledge, our meta-analysis is the first to compare double-dose/high-intensity statin monotherapy with moderate-intensity statin combined with ezetimibe dual therapy for lipid management in patients with T2DM. While no statistically significant difference was found in efficacy outcomes such as changes in LDL-C, HDL-C, total cholesterol, triglyceride levels, HBA1C, HOMA-IR, and HOMA-B levels between the two groups, of note, monotherapy demonstrated a lower risk of serious clinical adverse events but trended towards a higher risk of medication discontinuation. It is noteworthy that although the outcomes were not statistically significant, this may be due to the fact that the outcomes might have been underpowered to adequately detect statistical significance.

Our findings are in contrast to some previous studies that have compared different statin regimens, indicating that adding ezetimibe to moderate-intensity statin therapy significantly enhances lipid-lowering efficacy compared with high-intensity statin therapy alone [25–28].

A recent meta-analysis compared the effectiveness of statin-ezetimibe combination therapy versus statin monotherapy in diabetic and nondiabetic individuals regarding CVD risk [29]. Their study differed from ours as they did not use solely double-dose statin dosage in the monotherapy group as ours did. Nonetheless, their results showed a significantly lower risk of CVD with ezetimibe-statin combination therapy compared with statin alone, both in diabetic (RR, 0.69) and nondiabetic (RR, 0.68) individuals. There was no significant difference in risk reduction between diabetic and nondiabetic subjects, but the analysis did not extensively examine lipid parameters. Similarly, the IMProved Reduction of Outcomes: Vytorin Efficacy International Trial trial highlights the significant benefits of adding ezetimibe to statin therapy, demonstrating reductions not only in first cardiovascular events but also in additional and total cardiovascular events over the study period [30]. The additional reduction in LDL-C levels with ezetimibe is probably due to the combined impact of targeting multiple pathways involved in lipid metabolism. While statins primarily act on hepatic cholesterol synthesis, ezetimibe blocks cholesterol absorption in the small intestine. This collective action often results in similar or enhanced LDL-C reduction compared with higher doses of statins alone. By achieving comparable clinical benefits with lower-intensity statin therapy, the risk of side effects with high-intensity therapy such as muscle symptoms and insulin resistance is minimized [31]. On the other hand, our study demonstrated comparable efficacy outcomes with monotherapy associated with a lower incidence of serious clinical adverse events in our study. In its favor, intensifying statin therapy offers a simpler and potentially more cost-effective approach, leveraging the potency and familiarity of statins. Previous analyses have associated the use of ezetimibe with adverse effects such as diarrhea, and there’s a heightened chance of liver enzyme levels rising when ezetimibe is taken alongside a statin [32].

Among patients at very high risk, only 22% of individuals who received high-intensity statin monotherapy, 21% who received statin plus ezetimibe, and 58% who received combination therapy with a proprotein convertase subtilisin/kexin type 9 (PCSK9) inhibitor were able to achieve the LDL-C goal indicated by the 2019 European Society of Cardiology/European Atherosclerosis Society guideline [33]. Although these statistics indicate efforts aimed at decreasing the risk of atherosclerotic cardiovascular disease, empirical evidence on the lowering of LDL-C in real-life situations may be considerably less effective. A retrospective research conducted in Europe, which included more than 14,000 patients, found that over 80% of them had LDL-C values exceeding 70 mg/dl, even though they were undergoing moderate to high-intensity statin therapy [34]. Remarkably, even though they did not reach the LDL-C target, the majority of patients did not undergo any modifications or revisions to their treatment program. Similarly, the data from the getting to an improved understanding of low-density lipoprotein cholesterol and dyslipidemia management registry in the USA revealed that only 14% of patients with atherosclerotic cardiovascular disease and LDL-C values ≥70 mg/dL received intensified lipid-lowering medication [35]. More precisely, the study found that statin intensification occurred in 6.3% of instances, ezetimibe addition in 4% of cases, and PCSK9 inhibitor addition in 2% of cases. Therefore, there remains a need to either intensify the statin dose or add a combination medication to patient’s regime and as both appear to be equally efficacious, the decision between monotherapy and dual therapy should be individualized, considering factors such as patient comorbidities, risk profile, tolerability, and treatment goals.

### Strengths and limitations

One of the key strengths of this meta-analysis is its comprehensive inclusion of seven RCTs, ensuring a high level of evidence and minimizing the risk of bias inherent in observational studies.

However, our study also has a few limitations. First, the smaller number of total studies may be underpowered to detect statistical significance, potentially resulting in statistically insignificant outcomes. Second, while most of our studies reported no heterogeneity, some outcomes did exhibit heterogeneity, which could introduce variability and affect the reliability of the results. We attempted to address this by performing sensitivity analysis to identify influential studies.

Third, there were inconsistencies in the reporting of data among the studies, which made conducting the meta-analysis challenging. Some studies reported changes in lipid parameters as mean differences, some as least square mean differences, and some as simple baseline and follow-up values of lipid parameters. We only included those studies reporting the outcome similarly for statistical rigor and minimized manual conversions. Future studies should aim for more standardized reporting of outcomes.

Finally, the short duration of follow-up in the included studies may not capture the long-term effects and outcomes associated with different treatment regimens.

### Conclusions

In conclusion, our meta-analysis demonstrated comparable efficacy of double-dose intensity statin therapy versus combination therapy with moderate-intensity statin therapy and ezetimibe in patients with diabetes. While both treatments managed lipid levels and markers of insulin resistance comparably, monotherapy had a lower risk of serious clinical adverse events. Further research, including larger RCTs with longer follow-up periods and standardization of data reported and effect measures used to describe the changes is needed to clarify the clinical implications and guide optimal management strategies for this patient population.
